# Sediments Microfungi Diversity in the Demänovská Ice Cave: A Combined Morpho-Molecular Approach with Edaphic N, P, K, C and pH Factors

**DOI:** 10.3390/ijms27177812

**Published:** 2026-08-31

**Authors:** Rafał Ogórek, Magdalena Cal-Smok, Klaudyna Spychała, Agnieszka Lejman, Jakub Suchodolski

**Affiliations:** 1 Department of Mycology and Genetics, University of Wrocław, Przybyszewskiego Street 63-77, 51-148 Wrocław, Poland; magdalena.cal@uwr.edu.pl (M.C.-S.); klaudyna.spychala@uwr.edu.pl (K.S.); jakub.suchodolski@uwr.edu.pl (J.S.); 2 Institute of Agroecology and Plant Production, Wrocław University of Environmental and Life Sciences, 53-363 Wrocław, Poland; agnieszka.lejman@upwr.edu.pl

**Keywords:** subterranean ecology, soil, sediment, edaphic factors, micromycetes

## Abstract

Subterranean environments represent extreme oligotrophic ecosystems where microclimatic and edaphic factors restrict microbial life. This study evaluated culturable fungal communities in the soil and sediment samples of the Demänovská Ice Cave to determine their spatial diversity and abundance across psychrophilic (5 °C) and mesophilic (24 °C) incubation temperatures in relation to soil and sediment chemistry. Using a combined morpho-molecular approach, 19 distinct fungal species were identified, with a strong taxonomic dominance of Ascomycota and the genus *Penicillium*. Culturing temperatures acted as a primary selective filter. At 5 °C, specialized cold-adapted fungi (*Cladosporium cladosporioides* and *Pseudogymnoascus*) were more abundant in subterranean sediments than in surface samples. Conversely, incubation at 24 °C reduced fungal abundance and diversity in subterranean samples, with most fungal growth observed in surface samples from the outdoor control site. Statistical analysis revealed that at 24 °C, fungal abundance correlated significantly with total nitrogen (r = 0.909), available potassium (r = 0.878), and total organic matter (r = 0.757). However, under psychrophilic conditions, these nutrient-driven relationships were no longer observed, yielding non-significant interactions inside the cave. These findings demonstrate that subterranean microclimatic stability, rather than localized nutrient abundance, determines the ecological and distribution dynamics of ice cave mycobiota. In addition, cave sediments and soils can harbor opportunistic and toxigenic pathogens, including genera typically associated with azole resistance.

## 1. Introduction

Caves are subterranean ecosystems typically characterized by restricted inputs of organic and inorganic matter. Biological processes within these oligotrophic environments are largely shaped by relatively stable physicochemical parameters, including the permanent absence of light, high relative humidity, and low temperatures with minor seasonal fluctuations [1,2]. Consequently, cave mycobiota heavily depend on periodic allochthonous inputs from the surface [3,4], and may include potentially pathogenic taxa introduced by visitors in tourist-accessible caves [3,4,5,6,7].

Despite the limited energy availability, subterranean environments host diverse fungal communities, which contribute significantly to organic matter cycling, substrate biodeterioration, and biogeochemical transformations [5]. Therefore, cave microbiota are increasingly investigated for bioprospecting, as such extreme, selective conditions often favor the evolution of specialized strains producing novel enzymes and secondary metabolites with industrial and pharmaceutical applications [8,9].

Ice caves provide an exceptional setting for examining the ecology of culturable fungi within soil and sediment samples, as low temperature acts as a primary environmental filter governing fungal growth, physiological adaptation, and survival [10,11]. This ecological filtering is especially present in tourist-accessible ice caves, where a perennial cold microclimate overlaps with constant visitor pressure [10,12] and the Demänovská Ice Cave represents a model system for studying these interactive stressors [13,14]. While airborne fungi [12] and keratinophilic and keratinolytic fungal communities [15] have previously been documented within this specific cave, the functional dynamics of the soil-sediment interface remain poorly understood. Furthermore, the cave serves as an important habitat for bats, which act as biological carriers of microorganisms and provide concentrated organic matter in the form of guano, locally supporting fungal growth [16,17]. Subterranean sediments serve as long-term reservoirs for fungal propagules, integrating external inputs from air, water, humans, and cave-dwelling animals with local edaphic variations. Consequently, analyzing the soil-sediment mycobiota provides a more robust, long-term overview of fungal diversity and community stability [18] than highly seasonal and variable aeromycological samples [12].

Subterranean systems often exhibit complex spatial gradients. While surface environments are heavily enriched with macronutrients and organic matter, cave interiors typically feature high organic depletion, transitioning from extreme oligotrophy in outermost zones to localized organic accumulations in the furthest interior reaches [18]. Specialized cave-adapted mycobiota demonstrate a remarkable evolutionary capacity to survive and colonize under these extreme nutrient restrictions, often exhibiting a complete statistical decoupling from local substrate richness and species richness partition across localized microenvironments, such as cave transition and deep interior zones [18,19,20,21,22].

To map these complex dynamics and isolate functional ecotypes, speleomycological surveys commonly apply multi-temperature incubation strategies [23]. This approach serves as both a selective isolation method and an ecological diagnostic tool to differentiate true psychrophilic or psychrotolerant subterranean specialized species, such as *Cladosporium* and *Pseudogymnoascus*) from transient mesophilic or thermotolerant surface contaminants [18,24]. Importantly, the latter fractions frequently harbor opportunistic pathogens of medical, veterinary, and agricultural concern, including azole-resistant strains, dermatomycotic agents, allergenic bioaerosols, and toxigenic molds capable of synthesizing hazardous mycotoxins (like patulin and cyclopiazonic acid) via specialized secondary biosynthetic gene clusters [18,25,26,27,28].

To address these interconnected ecological, environmental, and public health aspects, the main objective of this study was to comprehensively assess the micromycetes communities inhabiting the soil and sediment samples of the Demänovská Ice Cave. Specifically, the study aimed to evaluate: (1) their spatial diversity across multiple sampling locations, (2) their abundance and taxonomic structure at different thermal incubation thresholds, and (3) the influence of selected edaphic factors on these subterranean communities. Importantly, this work extends previous research that focused exclusively on keratinophilic and keratinolytic fungi [15], as well as aeromycological surveys within this specific cave system [12]. By integrating the previously unexplored soil-sediment interface with the overall species pool and linking these biological data to soil chemistry, this study fills a key knowledge gap in subterranean ecology of this cave.

## 2. Results

### 2.1. Overview of Fungal Sampling

**Figure 1:**
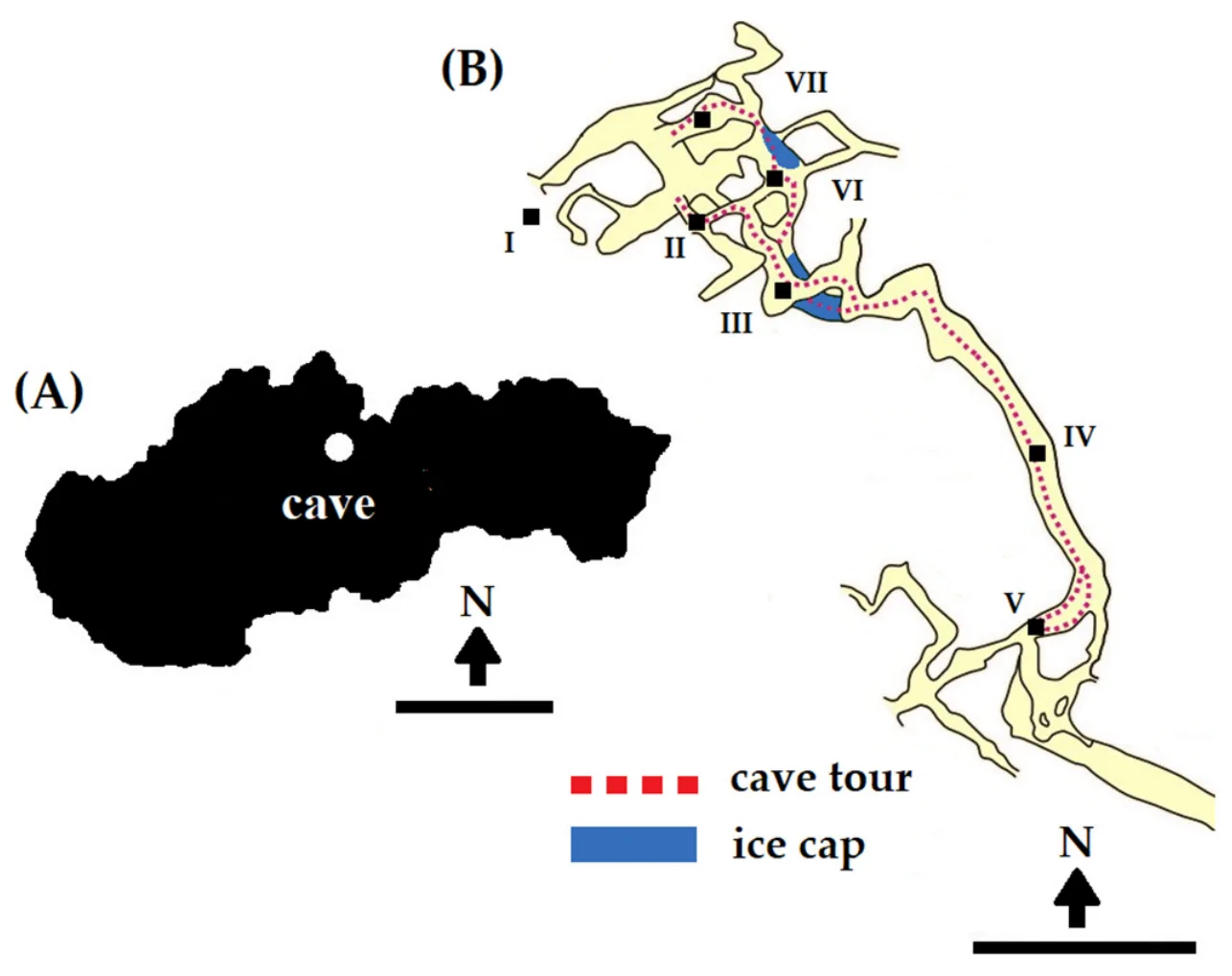
The regional positioning of the cave system within Slovakia (**A**). The detailed subterranean plan of the Demänovská Ice Cave (**B**), delineating the marked sampling sites (Sites I–VII), the established tourist route, and perennial ice-bearing sections. Sampling site I represents the outdoor surface control location situated outside the cave entrance, whereas sites II through VII are distributed sequentially within the cave interior along the visitor pathway. Scale bars: (**A**) = 100 km, (**B**) = 100 m.

To determine the diversity and concentration of culturable fungi in the Demänovská Ice Cave, soil and sediment samples from seven sampling sites were analysed. The material included one sample collected outside the cave (no. I) and six samples collected inside the cave (from no. II to no. VII) (Figure 1). Fungi were cultured on PDA medium at 5 ± 0.5 °C and 24 ± 0.5 °C.

### 2.2. Taxonomic Classification and Molecular Identification

The taxonomic classification, molecular identification metrics, and thermal distribution patterns of the fungal isolates recovered from the Demänovská Ice Cave are detailed in Table 1. Molecular sequencing of the internal transcribed spacer (ITS) region yielded fragments ranging from 351 to 582 base pairs (bp). BLAST (BLAST+ 2.17.0) analysis demonstrated an exceptionally high fidelity of taxonomic identification; all isolates achieved 100% query coverage and sequence identity scores ranging from 99.05% to 100.00% against reference sequences in the GenBank database (Table 1).

A total of 19 distinct fungal species (isolates UWR_1170 to UWR_1188; GenBank accession nos. PZ770540-PZ770558) were successfully isolated. The community exhibited a strong taxonomic dominance of the phylum Ascomycota (17 species, 89.5%), complemented by two zygomycetous representatives belonging to the phylum Mortierellomycota: *Linnemannia elongata* (UWR_1176) and *Mortierella alpina* (UWR_1177). At the genus level, the community was heavily characterized by *Penicillium*. This genus comprised nearly one-third of the total documented biodiversity with six identified species: *P. camemberti* (UWR_1179), *P. chrysogenum* (UWR_1180), *P. commune* (UWR_1181), *P. echinulatum* (UWR_1182), *P. griseofulvum* (UWR_1183), *P. restrictum* (UWR_1184), and *P. swiecickii* (UWR_1185)—Table 1.

### 2.3. Substrate Physicochemical and Nutrient Profiles

Across all examined study sites, the substrates exhibited relatively constant alkaline properties, with pH values ranging from 8.32 to 8.62 in H_2_O and 7.82 to 8.53 in KCl. The highest pH levels were documented at the innermost location (Site VII; pH in H_2_O = 8.62), while the subterranean average (pH in H_2_O = 8.42) aligned closely with the outdoor control point (Site I; pH in H_2_O = 8.47) (Table 2).

In contrast, a variation in organic content was observed regarding the organic components between the exterior and interior of the cave. The outdoor environment (Site I) was heavily enriched with organic carbon (OC = 5.02%) and total organic matter (8.65%). In contrast, the cave interior (Sites II–VII) was characterized by a high decrease in organic material, averaging just 1.28% for OC and 2.21% for organic matter. Within the subterranean ecosystem, a gradient was visible: the outermost cave zones (Sites II and III) were extremely oligotrophic (≤0.57% total organic matter), whereas the furthest interior site (Site VII) showed a accumulation of organic deposits, reaching 4.03% %OC and 6.95% organic matter. The macronutrient profile followed a similar spatial polarization. Total nitrogen (total N) was higher outside the cave (Site I; 1.84 g·kg^−1^) than the subterranean average (0.51 g·kg^−1^), reflecting the lack of direct surface vegetation inputs underground. Similarly, available potassium (total K) peaked outside the cave (90 mg·kg^−1^), being more than twofold higher than the average interior baseline (37.50 mg·kg^−1^). Conversely, total phosphorus (total P) demonstrated an inverse distribution pattern; the lowest phosphorus concentration was recorded at the outdoor site (20.60 mg·kg^−1^), whereas the subterranean sites showed consistently higher accumulation, averaging 30.68 mg·kg^−1^ and peaking at Site VII (41.53 mg·kg^−1^) (Table 2).

### 2.4. Fungal Species Richness and Alpha Diversity

**Figure 2:**
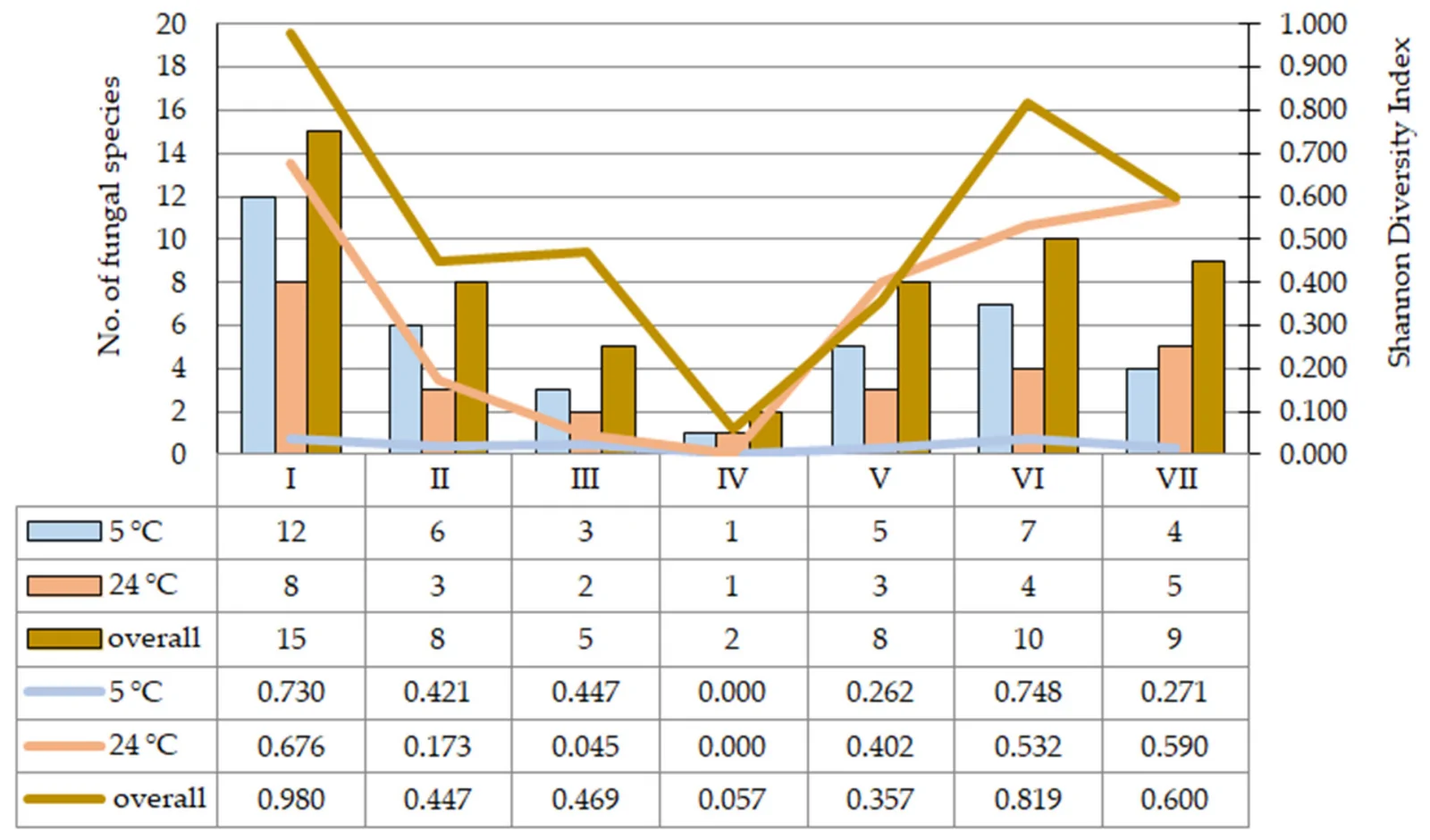
Fungal species richness and Shannon Diversity Index (H) values across individual sampling locations Fungal species richness is represented by the vertical columns, whereas the corresponding alpha diversity metrics (H) are indicated by the continuous lines. Metrics were calculated for soil and sediment samples collected across the designated sampling array, which includes the outdoor control location (Site I) and the subterranean interior locations (Sites II–VII) of the Demänovská Ice Cave.

The data from the Demänovská Ice Cave revealed a variation in both the absolute number of isolated species and the corresponding Shannon Diversity Index (H) values across different incubation thermal thresholds (Figure 2). Under psychrophilic incubation conditions (5 °C), the outdoor control point (Site I) harbored the highest species richness with 12 species (H = 0.730). Within the subterranean environment, a gradient was observed; fungal diversity was the highest at the transition zone at site VI (7 species, H = 0.748) and site II (6 species, H = 0.421). In contrast, site IV exhibited extreme biological restriction, yielding only a single isolated species and a completely suppressed diversity index (1 species, H = 0.000). The remaining cave sites maintained low to moderate richness, ranging from 3 species at site III (H = 0.447) to 5 species at site V (H = 0.262).

Incubation at a higher mesophilic threshold (24 °C) induced severe biodiversity suppression across the majority of the subterranean locations, while the outdoor site remained relatively rich (8 species, H = 0.676). Inside the cave, species counts decreased to a range of 1 to 5 species. The most profound constraint was observed at site III (2 species, H = 0.045) and site IV (1 species, H = 0.000). Interestingly, a minor increase in mesophilic diversity was noted in the deeper cave zones, specifically at site VII (5 species, H = 0.590) and site VI (4 species, H = 0.532), whereas site II and site V dropped to 3 species each, with H values of 0.173 and 0.402, respectively (Figure 2).

The cumulative overall analysis across both temperature thresholds confirmed the outdoor location (Site I) as the main source of mycobiota biodiversity, pooling a total of 15 species and the highest diversity index (H = 0.980). Within the cave system, site VI emerged as the most significant subterranean hotspot, accumulating a total of 10 species (H = 0.819), followed closely by site VII (9 species, H = 0.600) and site II (8 species, H = 0.447). Site IV remained clearly established as the most oligotrophic and low-diversity zone within the entire study area, accumulating only 2 species and a critical overall diversity baseline (H = 0.057)—Figure 2.

### 2.5. Effects of Culturing Temperature and Spatial Partitioning

**Figure 3:**
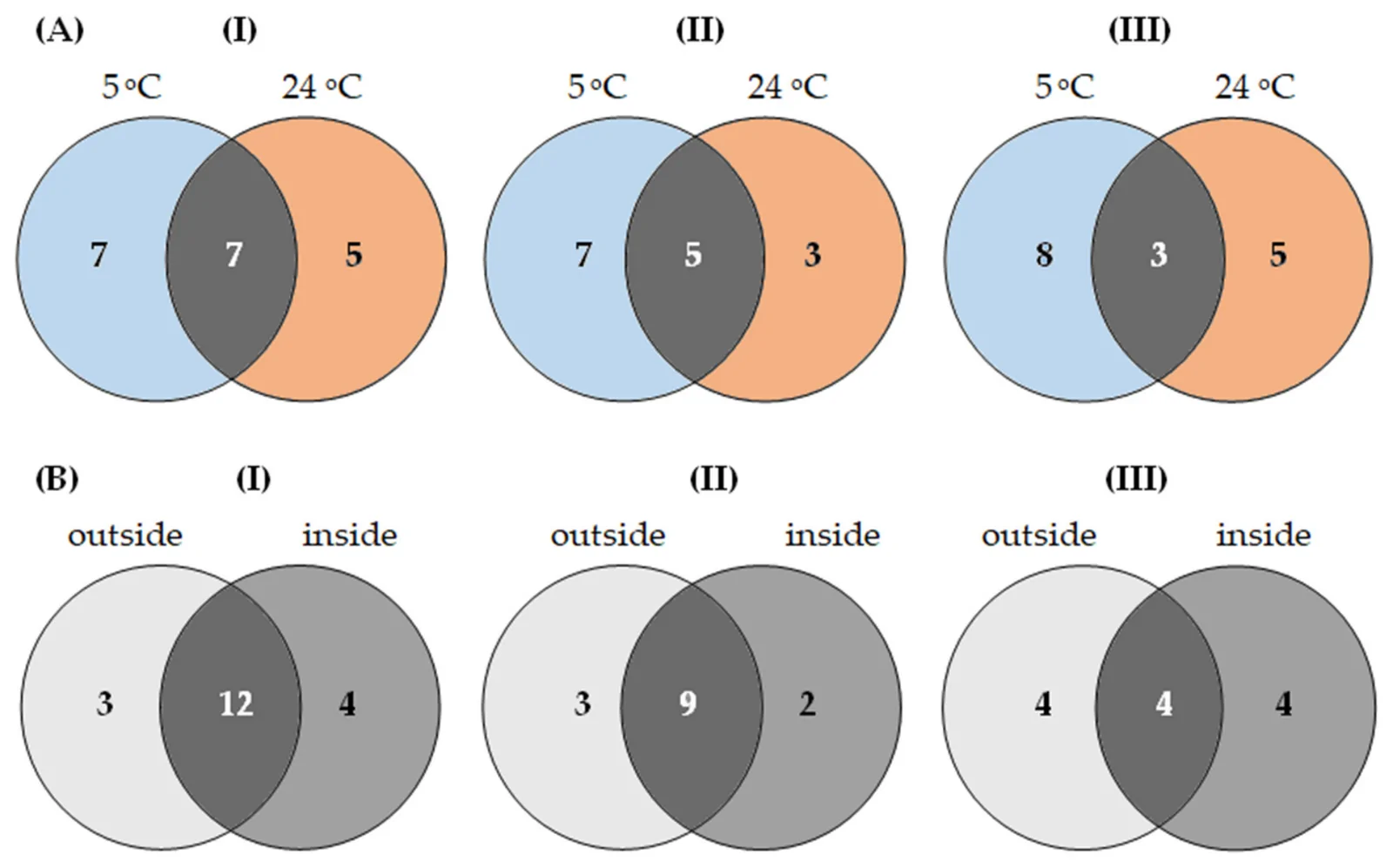
Effects of incubation temperature and spatial partitioning on fungal species distribution. Panel (**A**) illustrates the isolated effect of incubation temperature on the number of recovered fungal species, categorized without study site differentiation (**A**(**I**)), strictly within the outdoor surface control samples (**A**(**II**)), and within the subterranean cave interior samples (**A**(**III**)). Panel (**B**) illustrates the direct effect of geographic study sites on fungal species distribution, categorized without thermal incubation differentiation (**B**(**I**)), under a psychrophilic regime at 5 °C (**B**(**II**)), and under a mesophilic regime at 24 °C (**B**(**III**)).

The isolated and interactive effects of incubation temperature and geographical partitioning (outside versus inside the cave environment) on the sharing and distribution of the 19 identified fungal species are illustrated in Figure 3. as delineated in Panel A, when analyzing the entire fungal collection without study site differentiation (Figure 3A(I)), a total of 19 distinct species were resolved; psychrophilic cultivation (5 °C) yielded 14 species (7 unique and 7 shared), whereas mesophilic cultivation (24 °C) yielded 12 species (5 unique and 7 shared). When assessing the outdoor control location independently (Figure 3A(II)), the community comprised 15 species (3 unique mesophilic species and 7 unique psychrophilic species); moreover, 5 species overlapped between both temperatures. Conversely, within the isolated subterranean environment (Figure 3A(III)), cold-adapted preferences became prominent within the 16 total inside species, where low-temperature cultivation (5 °C) supported 8 unique species, while the warmer threshold (24 °C) supported 5 unique species, leaving only 3 species capable of growth across both thermal regimes (Figure 3A).

The spatial influence evaluated across incubation temperatures is summarized in Panel B. When temperature parameters were completely pooled (Figure 3B(I)), the outdoor environment and the cumulative cave interior exhibited a high degree of taxonomic overlapping, sharing 12 species out of the total 19, with 3 species being strictly exclusive to the outside and 4 species restricted to the inside. Under specific cold incubation at 5 °C (Figure 3B(II)), out of 14 total species, 9 were shared between both habitats, while 3 were exclusive to the outside and 2 were found only inside the cave. In contrast, under the mesophilic regime at 24 °C) (Figure 3B(III)), the distribution became perfectly symmetrical across the 12 active species; the outdoor exclusive pool, the shared pool, and the subterranean interior exclusive pool each contained exactly 4 species (4 unique outside, 4 shared, and 4 unique inside) (Figure 3B).

### 2.6. Quantitative Biomass Accumulation

**Figure 4:**
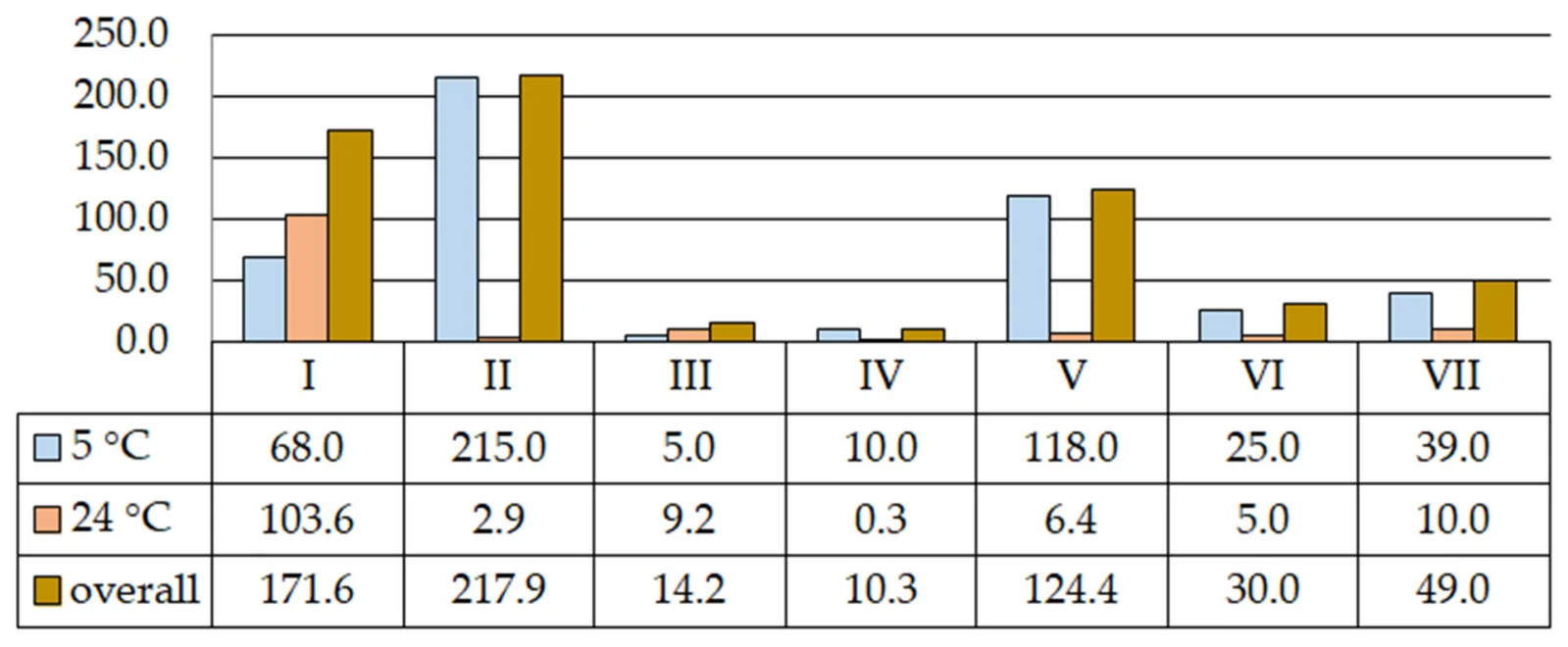
The culturable fungal counts (CFU·10^2^ g^−1^) across individual sampling locations, featuring the outdoor surface control location (Site I) and the subterranean interior locations (Sites II–VII) of the Demänovská Ice Cave following incubation at psychrophilic (5 °C) and mesophilic (24 °C) thresholds.

Quantitative recovery of culturable fungi (CFU·10^2^ g^−1^; Figure 4) demonstrated a clear, temperature-dependent physiological divergence between the outdoor control (Site I) and subterranean environments (Sites II–VII). Under psychrophilic incubation (5 °C), subterranean sediments revealed a proliferation capacity that outperformed the surface baseline. Fungal counts inside the cave peaked at Site II (215.0 CFU·10^2^ g^−1^), followed by Site V (118.0 CFU·10^2^ g^−1^), while the outdoor control maintained a moderate density of 68.0 CFU·10^2^ g^−1^. The remaining cave locations exhibited severe biological restriction, down to a minimum of 5.0 CFU·10^2^ g^−1^ at Site III.

Conversely, mesophilic incubation (24 °C) inverted this quantitative distribution. The outdoor community (Site I) exhibited dominance, surging to 103.6 CFU·10^2^ g^−1^, whereas all subterranean locations underwent a biomass collapse. Fungal counts underground dropped below 10.0 CFU·10^2^ g^−1^ across all cave sites, reaching a base minimum at Site IV (0.3 CFU·10^2^ g^−1^). Cumulative values (combined thermal regimes) confirmed Site II (217.9 CFU·10^2^ g^−1^), Site I (171.6 CFU·10^2^ g^−1^), and Site V (124.4 CFU·10^2^ g^−1^) as the primary ecological hotspots, while Site IV (10.3 CFU·10^2^ g^−1^) remained conclusively established as the most depleted, oligotrophic zone (Figure 4).

### 2.7. Standardized Spatial Performance Assessment (z-Scores)

The descriptive statistics, spatial deviations, and standardized values (*z*-scores) for fungal CFU across the soil and sediment samples of the Demänovská Ice Cave are comprehensively summarized in Table A1, Table A2 and Table A3. The baseline reference parameters exhibited substantial fluctuations depending on the incubation temperature. Culturing at 5 °C yielded a higher mean fungal abundance (M = 68.6 CFU, SD = 75.5 CFU; Table A1) compared to incubation at 24 °C (M = 19.6 CFU, SD = 37.17 CFU; Table A4). When evaluating the combined data across both incubation temperatures, the baseline parameters culminated in an overall mean (M) of 88.2 CFU and a standard deviation (SD) of 83.23 CFU (Table A3).

The analysis revealed fungal distribution patterns between the outdoor control point (Site I) and the subterranean locations (Sites II–VII). At the psychrophilic/psychrotolerant incubation threshold (5 °C), the highest fungal abundance was documented inside the cave at study site II (*z* = 1.94, 97.4th percentile), followed by a moderate above-average trend at site V (z = 0.66, 74.4th percentile). Interestingly, the outdoor location (Site I) aligned almost perfectly with the norm baseline under these cold conditions (*z* = −0.01, 49.7th percentile).

In contrast, mesophilic incubation at 24 °C altered the community distribution, establishing the outdoor location (Site I) as a critical outlier with an exceptionally high raw score of 103.6 CFU (*z* = 2.26), positioning it within the 98.8th percentile. Conversely, all cave interior locations (Sites II–VII) experienced growth inhibition at 24 °C clustering uniformly below the baseline average within a narrow margin between the 30th and 40th percentiles.

The combined temperature evaluation (Table A3) integrates these dynamics, highlighting that both the outdoor site (Site I; *z* = 1.00, 84.2nd percentile) and the cave interior site II (*z* = 1.56, 94.0th percentile) represent the primary hotspots for fungal abundance, though driven by different thermal ecotypes. The remaining subterranean sites (III, IV, VI, and VII) consistently demonstrated a restricted fungal presence across all monitoring series, with the lowest overall relative performance observed at site IV under the combined parameters (*z* = −0.9), 17.5th percentile.

### 2.8. Taxonomic Structure and Community Composition

**Figure 5:**
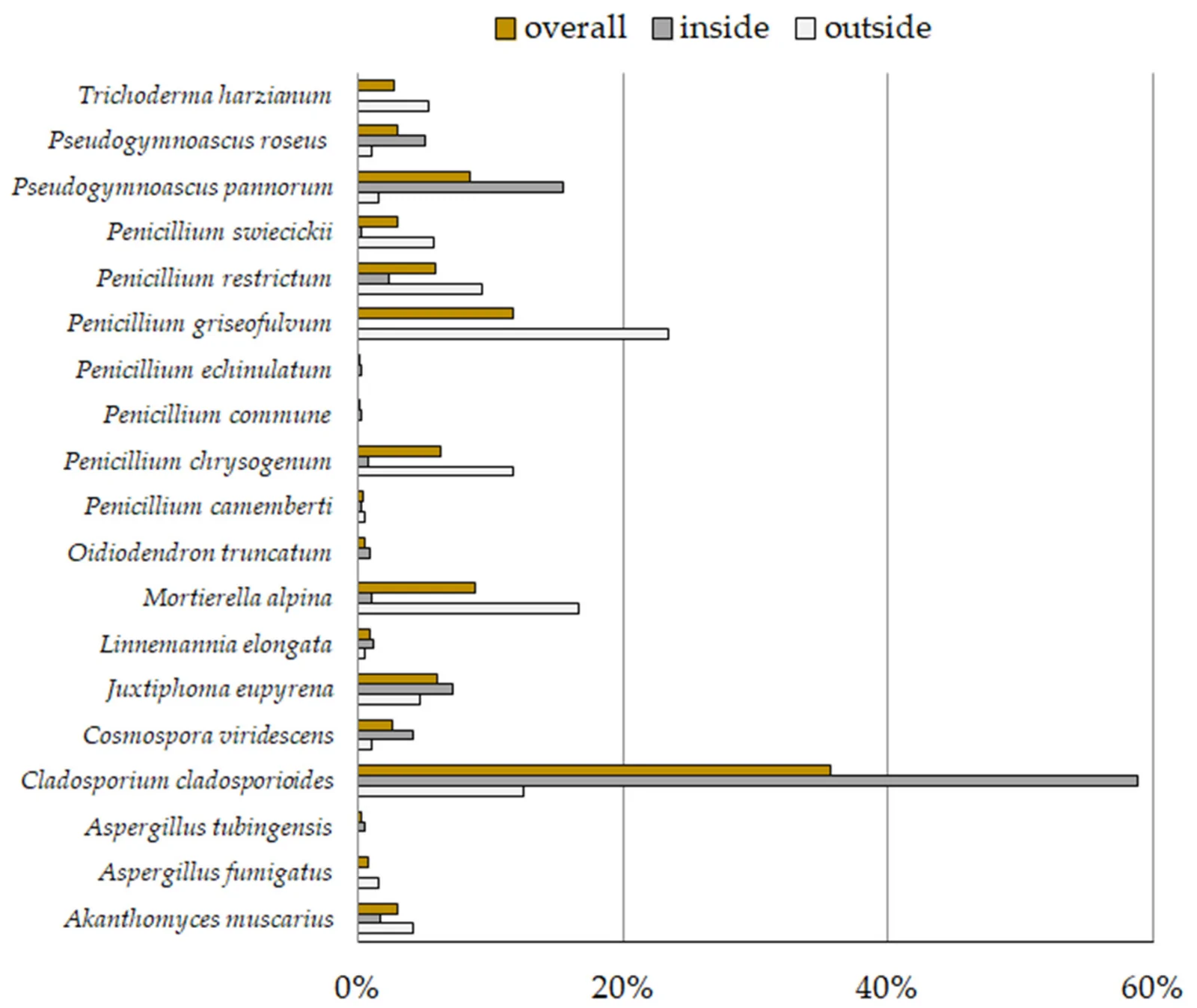
Overall values represent the cumulative sum of CFU·10^2^ g^−1^ obtained across both incubation temperatures for each individual site of the Demänovská Ice Cave. The percentage contribution of individual fungal taxa to the total pool of culturable fungi.

The taxonomic structure and percentage contribution of isolated fungal species outside and inside the cave are detailed in Figure 5, Table A4 and Table A5. In the outdoor control environment (outside), the community was primarily dominated by *P. griseofulvum* (23.49%), *M. alpina* (16.60%), *Cladosporium cladosporioides* (12.53%), and *P. chrysogenum* (11.74%). Conversely, the cave interior (inside) exhibited a taxonomic shift, where *C. cladosporioides* became overwhelmingly dominant, accounting for 58.91% of the entire subterranean mycobiota. The overall combined dataset (overall) integrated these distinct habitats, reflecting a general dominance of *C. cladosporioides* (35.72%), *P. griseofulvum* (11.74%), *M. alpina* (8.80%), and *P. pannorum* (8.50%), while the remaining 15 species individually contributed minor fractional shares below 6.3% (Figure 5).

Fungal species distribution across thermal regimes (Table A4 and Table A5) revealed a strict physiological segregation between habitats. At 5 °C (Table A1), high subterranean biomass was predominantly driven by *C. cladosporioides*, which peaked at Site II (152.0 × 10^2^ CFU g^−1^) and Site V (100.0 × 10^2^ CFU g^−1^). In contrast, deeper oligotrophic zones (Sites III, IV, VII) were characterized by a transition toward the genus *Pseudogymnoascus*, with *P. pannorum* acting as the sole species at the restricted Site IV (10.0 × 10^2^ CFU g^−1^) and reaching maximum density at Site VII (24.0 × 10^2^ CFU g^−1^).

Conversely, incubation at 24 °C (Table A5) induced community turnover, restricting fungal proliferation primarily to the outdoor control (Site I). This surface surge was co-dominated by *P. griseofulvum* (45.0 × 10^2^ CFU g^−1^), *M. alpina* (30.3 × 10^2^ CFU g^−1^), *C. cladosporioides* (20.0 × 10^2^ CFU g^−1^), and *P. chrysogenum* (20.0 × 10^2^ CFU g^−1^). Within the cave interior, these mesophilic taxa decreased, leaving the remaining sparse subterranean community largely dependent on *Cosmospora viridescens* (e.g., at Site III and V), while true cave specialists like *Pseudogymnoascus* spp. were completely inhibited.

### 2.9. Environmental Correlations and Substrate Drivers

**Figure 6:**
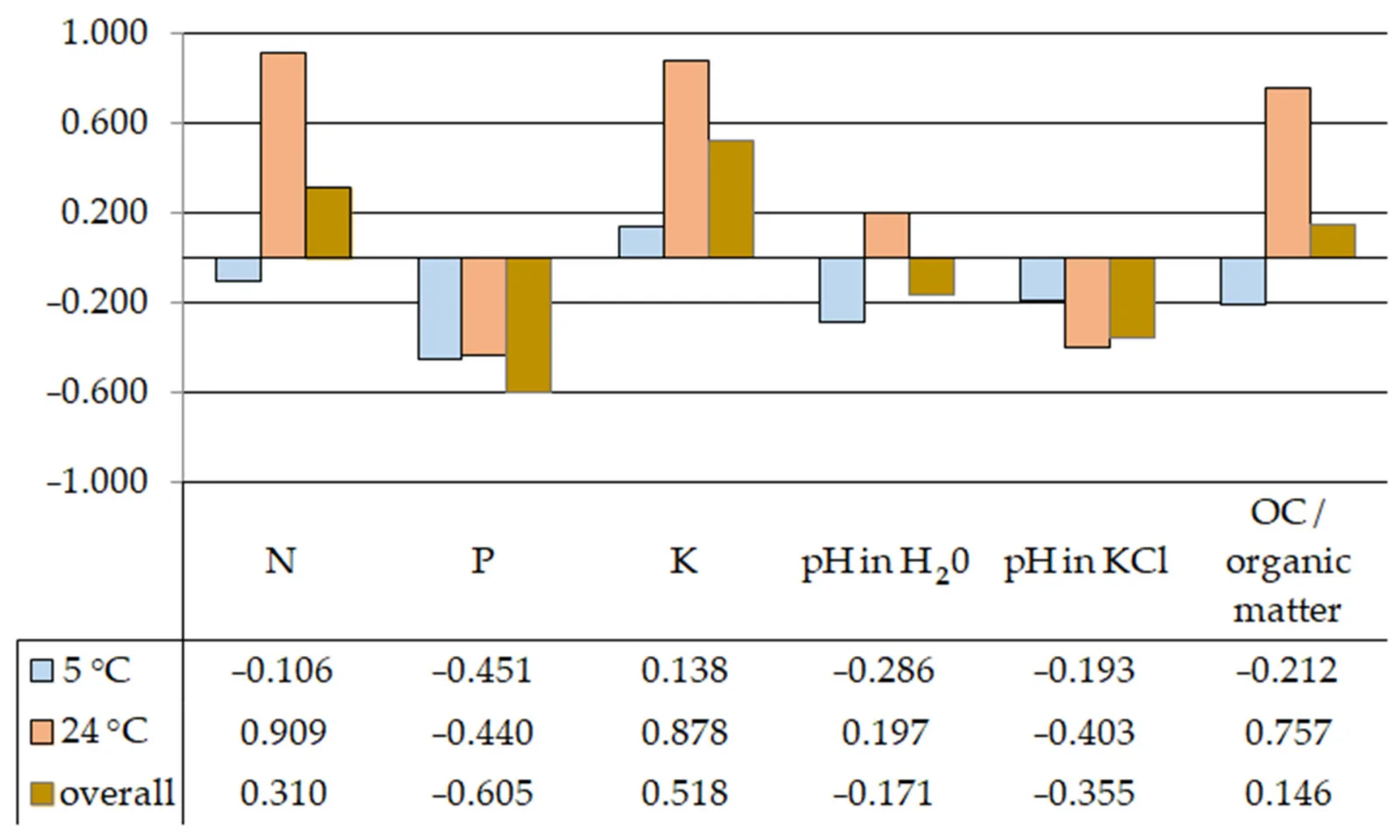
Pearson Correlation Coefficients (*r*) between culturable fungal abundance and substrate physicochemical properties. Fungal counts were obtained from soil and sediment samples of the Demänovská Ice Cave following incubation at psychrophilic (5 °C) and mesophilic (24 °C) thermal thresholds, alongside the cumulative overall values pooled for both temperatures. Environmental parameters include edaphic nutrient concentrations (total nitrogen, total phosphorus, available potassium), organic carbon (%OC), total organic matter (%), and pH values.

The Pearson correlation coefficients (*r*) and corresponding significance levels (p) between culturable fungal abundance and substrate physicochemical properties across distinct thermal incubation regimes are illustrated in Figure 6. The statistical analysis revealed highly contrasting, temperature-dependent correlation patterns between the outdoor and subterranean environments.

At the mesophilic incubation threshold (24 °C), statistically significant positive correlations were observed between fungal abundance and total nitrogen (*r* = 0.909, *p* = 0.005), available potassium (*r* = 0.878, *p* = 0.009), and total organic matter content (*r* = 0.757, *p* = 0.049). In contrast, the remaining substrate relationships under this threshold showed noticeable trends but failed to reach statistical significance, including a moderate negative association with total phosphorus (r = −0.440, *p* > 0.05) and weak interactions with pH measured in pH in H_2_O (*r* = 0.197, *p* = 0.673) and pH in KCl (*r* = −0.403, *p* = 0.370)—Figure 6.

Under psychrophilic conditions (5 °C), these positive nutrient-driven relationships completely decoupled, yielding weak and statistically non-significant interactions across all analyzed edaphic parameters. Fungal density under cold conditions exhibited minor negative trends with total nitrogen (r = −0.106, *p* = 0.820), total organic matter (*r* = −0.212, *p* = 0.648), total phosphorus (*r* = −0.451, *p* = 0.310), and pH in KCl (*r* = −0.193, *p* = 0.678), alongside weak associations with available potassium (*r* = 0.138, *p* = 0.767) and pH in H_2_O (*r* = −0.286, *p* = 0.536)—Figure 6.

When the dataset was pooled globally without separation into specific incubation temperatures, no statistically significant relationships were detected. The cumulative overall analysis reflected weak to moderate, non-significant correlations for total nitrogen (*r* = 0.310, *p* = 0.499), available potassium (*r* = 0.518, *p* = 0.433), organic carbon/organic matter (*r* = 0.146, *p* = 0.819), pH in H_2_O (*r* = −0.171, *p* = 0.819), and pH in KCl (*r* = −0.355, *p* = 0.613). Notably, total phosphorus demonstrated the strongest cumulative trend with a pronounced but non-significant negative correlation (*r* = −0.605, *p* = 0.150) against the pooled fungal counts—Figure 6.

## 3. Discussion

The spatial distribution and quantitative recovery of micromycetes across the seven sampling sites in the Demänovská Ice Cave demonstrate a high degree of compartmentalization between the surface and subterranean sites. The selection of PDA medium and dual incubation temperatures (5 °C and 24 °C) proved crucial for capturing the metabolic dichotomy of the ecosystem, a methodological approach validated in similar studies of underground environments [15]. The identification of a single outdoor control point (Site I) and six distinct cave locations (Sites II–VII) allowed for a robust evaluation of how the physical topography of the cave influences fungal deposition. The absolute dominance of surface biomass at 24 °C versus the localized explosion of cold-adapted biomass inside the cave at 5 °C highlights that subterranean sediments do not merely act as passive repositories, but as active selective boundaries for specific mycobiota fractions, as observed in other Carpathian caves [17,18].

The taxonomic structure resolved ITS sequencing showed a clear dominance of the phylum Ascomycota (89.5%) reflects global subterranean data, where this group consistently outcompetes others due to robust spore survival and diverse enzymatic capabilities [21,29]. At the genus level, the heavily documented footprint of *Penicillium* (nearly one-third of the total biodiversity) is highly characteristic of environmental samples, including underground ecosystems [18,28,30,31]. *Penicillium* species possess hyper-stable sporulation kinetics and can withstand low water activity and severe nutrient restriction [31]. However, the isolation of specific zygomycetous representatives such as *L. elongata* and *M. alpina* adds an important ecological nuance. These species are typically functional saprotrophs in cold terrestrial soils, and their presence inside the cave indicates a strong phylogenetic tie to the surrounding forest soils, from which they are likely transported via water infiltration and air masses [32].

The constant alkaline properties observed across all study sites (pH ranging from 8.32 to 8.62 in H_2_O) represent a stable chemical baseline typical of limestone-based karstic networks [33]. In contrast, the dramatic polarization of organic matter content between the exterior and interior zones reveals a classic allochthonous-dependent ecosystem. The outdoor environment (Site I) is highly enriched with organic carbon (5.02%) and total organic matter (8.65%) probably due to direct litter fall and vegetation inputs. Deep inside the cave, a sharp depletion is visible, averaging just 1.28% OC. Interestingly, the accumulation of organic deposits at the furthest interior site (Site VII; 4.03% OC) presents a localized anomaly. This gradient suggests that Site VII acts as a geological trap or sedimentary sink, where microclimatic airflows, humidity condensation, or gravitational water dripping focus organic remains that cannot escape the cave system [19,34]. The polarization of total nitrogen (1.84 g·kg^−1^ outside vs. 0.51 g·kg^−1^ inside) further confirms that subterranean microfungi must execute their life cycles under severe oligotrophic limitations unknown to surface species [21].

The variations in the Shannon Diversity Index (H) and absolute species richness across different thermal thresholds clearly establish temperature as the primary filter. Under psychrophilic conditions (5 °C), the cave transition zones (Site VI, 7 species, H = 0.748; Site II, 6 species, H = 0.421) emerged as biological hotspots. These locations experience mild airflow transitions and seasonal fluctuations, allowing for the stable co-existence of both transient surface spores and stable, cold-adapted cave specialists, a phenomenon extensively documented by Docampo et al. [35]. Conversely, the total suppression of diversity at Site IV (H = 0.000) across both temperature regimes confirms its status as an extremely isolated, ultra-oligotrophic core where ecological niche space is nearly non-existent [18,36,37,38]. Incubation at the mesophilic threshold (24 °C) induced severe biodiversity suppression across the cave interior, while the outdoor site remained relatively rich (8 species, H = 0.676). This structural collapse indicates that the vast majority of fungal propagules found deep inside the sediments are cold-preferring or cold-tolerant ecotypes, which face severe metabolic stress or permanent inhibition when exposed to elevated temperatures [29,32].

The interactive effects of temperature and geography highlight a strict physiological segregation. The asymmetrical distribution of the 19 identified species confirms that the outdoor and indoor pools operate on different environmental axes. At 5 °C, the fact that 9 species were shared between both habitats indicates that a significant portion of the surface mycobiota consists of psychrotolerant strains capable of surviving cave entry [20,21]. In contrast, the perfectly symmetrical distribution observed under the mesophilic regime at 24 °C (4 unique outside, 4 shared, and 4 unique inside) reveals a highly balanced community turnover. The 4 unique inside mesophilic species represent specialized, dormant propagules that remain metabolically inactive under natural cave conditions but can be activated when a thermal window opens [38]. The remaining sparse subterranean community’s reliance on *C. viridescens* at the colder sites (III and V) shows a specific ecological shift toward specialized micro-parasitic or opportunistic survival strategies, while true cave specialists like *Pseudogymnoascus* spp. are completely suppressed by mesophilic temperatures [15,32].

The clear divergence in quantitative biomass accumulation (CFU·10^2^ g^−1^) reinforces the concept of different thermal ecotypes dominating the surface and subterranean matrices. Under psychrophilic incubation, the cave sediments outperforming the surface baseline (peaking at Site II with 215.0 CFU) indicates that cold-adapted fungi find an ideal, stable ecological niche underground where surface competitors are absent [12,15]. The complete inversion of this pattern at 24 °C, where the outdoor community increased to 103.6 CFU while the cave interior collapsed below 10.0 CFU, confirms that true cave specialists are highly sensitive to thermal conditions [39]. This physiological vulnerability highlights that even minor microclimatic shifts (such as those induced by high tourist traffic, the presence of bats or external climate change) could lead to a rapid biomass collapse of the native, cold-adapted cave mycobiota, allowing surface-derived contaminants to permanently colonize the cave soil and sediments [6,19,23].

The standardized spatial performance analysis provides statistical backing for identifying genuine ecological hotspots. The high raw score and extreme *z*-score of the outdoor location at 24 °C (*z* = 2.26, 98.8th percentile) mathematically establishes Site I as a critical outlier driven by intense nutrient availability [28]. Under cold conditions, the alignment of Site II (z = 1.94, 97.4th percentile) and Site V (*z* = 0.66, 74.4th percentile) confirms that these specific transition zones are the primary drivers of subterranean fungal production [12]. The similar clustering patterns of all cave interior locations below the baseline average at 24 °C (between the 30th and 40th percentiles) shows a highly predictable, standardized growth inhibition across the entire subterranean ecosystem, proving that thermal constraints outperform local substrate differences when temperature thresholds are crossed [18].

The profound taxonomic shift from an outdoor community dominated by *P. griseofulvum* and *M. alpina* to a subterranean assembly overwhelmingly dominated by *C. cladosporioides* (58.91%) represents a classic paradigm of directional environmental selection. *C. cladosporioides* is widely known for its ability to dominate epilithic biofilms and air masses in ice caves due to its tolerance to low temperatures, high relative humidity, and to its high metabolic adaptability [20]. The transition toward *Ps. pannorum* as the sole survivor at the restricted Site IV (10.0 CFU) and its high density at Site VII emphasizes the deep evolutionary adaptation of true cave specialists. *Pseudogymnoascus* species are highly adapted psychrophiles that produce specialized enzymes capable of performing metabolic functions at near-freezing temperatures where typical mesophilic invaders remain completely dormant [32].

The most critical finding of this study is the complete absence of nutrient-driven statistical relationships under cold conditions. At the mesophilic threshold (24 °C), the significant positive correlations between fungal abundance and total nitrogen (r = 0.909), available potassium (r = 0.878), and total organic matter (r = 0.757) match standard terrestrial models where heterotrophic growth is directly fueled by substrate richness [31]. Environmental correlations under the actual cave psychrophilic baseline (5 °C), however, completely decoupled within the cave system, rendering weak and non-significant interactions across all analyzed edaphic variables (r = −0.106 for N; r = −0.212 for organic matter). This statistical decoupling highlights that specialized cave mycobiota (*Pseudogymnoascus* spp., *C. cladosporioides*) have evolved to operate entirely independently of localized nutrient concentrations. Their spatial distribution and growth dynamics are strictly dictated by microclimatic stability and low temperature conditions rather than nutrient wealth [32]. This adaptive independence allows specialized subterranean microfungi to maintain a stable ecological niche and drive essential biogeochemical cycles within the permanent ice and sediment matrices of the cave ecosystem.

Another aspect is the analysis of the obtained fungal communities in terms of their interaction with plants and living organisms. The highest pathogenic potential is exhibited by *A. fumigatus*, a ubiquitous, thermotolerant opportunistic pathogen and the primary etiological agent of invasive aspergillosis, which carries a high mortality rate in severely immunocompromised patients [40]. Conversely, *A. tubingensis* (section *Nigri*), although more frequently described as a saprotroph, shows increasing potential as an opportunistic pathogen [41]. More importantly, certain environmental isolates of this species display naturally reduced susceptibility to commonly used azole antifungicals, including voriconazole and posaconazole [25]. *P. pannorum* is a recognized etiological agent of superficial dermatomycoses and nail infections (onychomycosis) in humans, particularly in individuals with regular soil contact [26]. On the other hand, spores of *C. cladosporioides*, *P. chrysogenum*, and *P. commune* are permanent components of indoor bioaerosols. Their massive presence in the human environment induces type I hypersensitivity reactions, leading to the development of allergic asthma and hypersensitivity pneumonitis [27].

Isolates of *P. griseofulvum*, *P. echinulatum*, *P. restrictum*, and *P. swiecickii* synthesize hazardous mycotoxins, including patulin and cyclopiazonic acid [28]. In the context of phytopathology, *J. eupyrena* plays a critical role, causing seedling damping-off, root rot, and spot diseases in potato (*Solanum tuberosum*) and conifers [42]. *Akanthomyces muscarius* is a specialized entomopathogen that infects the insect cuticle (e.g., aphids, whiteflies) utilizing chitinases and proteases, ultimately leading to host death [43]. *Trichoderma harzianum* exhibits aggressive mycoparasitism, destroying the hyphae of soil-borne phytopathogens through lytic activity [44]. In contrast, *L. elongata* functions as a plant growth-promoting fungus (PGPF), optimizing hormonal balance and stimulating the above-ground biomass development of the host [45]. In turn, *M. alpina* is widely utilized in industrial biotechnology as a highly efficient producer of polyunsaturated fatty acids (PUFAs), including arachidonic acid [46].

## 4. Materials and Methods

### 4.1. Study Area

The study was conducted in the Demänovská Ice Cave (Demänovská ľadová jaskyňa), which is one of the most visited underground sites in Slovakia [47]. It is located on the right side of the Demänovská Valley, on the northern slope of the Low Tatras, Slovakia. The cave is situated within the Demänovská Valley National Nature Reserve in the Low Tatras National Park and forms the northern part of the Demänovský Cave System [13]. Its entrance is located below the Bašta cliff at an elevation of 840 m a.s.l., approximately 90 m above the valley bottom, at GPS coordinates N 49.0162010, E 19.5827999 [48]. The cave is 2445 m long and 57 m deep. The show path is 650 m long, with an elevation difference of 48 m. During the summer months, the air temperature in the cave ranges from +0.4 to +3.0 °C, and relative humidity reaches 92–98% [14].

The Demänovská Ice Cave is characterized by permanent ice accumulations, large underground chambers, and distinctive cave fauna. Ice accumulations occur in the lower parts of the cave. The cave also represents an important subterranean habitat. Eight bat species have been recorded there, and the cave is considered the largest wintering site of *Eptesicus nilssonii* and one of the most important wintering sites of *Myotis mystacinus*/*brandtii* in Slovakia [14].

### 4.2. Sampling

Soil and sediment samples were collected in 2022 from eight sampling sites from of the Demänovská Ice Cave (one outside the cave and seven inside it; Figure 1), according to the procedure described by Ogórek et al. [49]. Approximately 500 g of soil or sediment was collected from each site using sterile plastic scoops and placed in sterile bags. The samples were transported to the laboratory under refrigerated conditions (10 ± 2.0 °C) and stored at 5 ± 0.5 °C until mycological analysis, for no longer than 7 days.

### 4.3. Culture of Fungi from Samples

Before analysis, the samples stored under refrigerated conditions were aseptically mixed. Subsequently, 3 g of soil or sediment was transferred into individually wrapped and sterile polypropylene tubes (50 mL, with screw caps, FL Medical, Italy), suspended in 12 mL of sterile saline solution (0.85% NaCl), and shaken for 20 min at room temperature (24 ± 1 °C). The suspensions were diluted 10, 100, 1000 ×, then vortexed and 100 μL of each dilution was spread onto potato dextrose agar plates (PDA, BioMaxima, Lublin, Poland) in triplicate. The plates were incubated in darkness at 5 ± 0.5 °C and 24 ± 0.5 °C for 5 to 42 days, until no new fungal colonies appeared. Incubation at 5 °C was used to enable the isolation of psychrophilic and psychrotolerant fungi, and incubation at 24 °C was applied for mesophilic fungi. After incubation, fungal colonies were counted, and the average number of colony-forming units was expressed as CFU per gram (CFU·g^−1^) of soil or sediment. Fungal colonies were subcultured on PDA plates and incubated in darkness at the same temperature conditions for 4 to 35 days. After incubation, the isolates were purified using the single-spore method and maintained on PDA slants [50]. The obtained isolates were then subjected to phenotypic and molecular identification.

### 4.4. Fungal Identification

Individual fungal isolates were identified using both phenotypic and molecular methods. Preliminary phenotypic identification was based on macro- and microscopic observations of pure cultures grown on PDA. Colony colour, growth characteristics, pigmentation and the presence of diagnostic morphological structures were taken into account [18]. Phenotypic assessment was supported by diagnostic keys, monographs and taxonomic descriptions appropriate for the identified genera and species [51,52,53,54,55,56,57]. Molecular identification was performed based on the analysis of the internal transcribed spacer region of ribosomal DNA (ITS rDNA). Genomic DNA was isolated from fungal colonies using the Bead-Beat Micro AX Gravity kit (A&A Biotechnology, Gdańsk, Poland), according to the manufacturer’s protocol. ITS rDNA fragments were amplified using the primer pair ITS1 (5′-TCCGTAGGTGAACCTGCGG-3′) and ITS4 (5′-TCCTCCGCTTATTGATATGC-3′) [58]. PCR was performed in a T100 Thermal Cycler (Bio-Rad), in accordance with the method previously described by Ogórek et al. [17]. The PCR products were visualized by electrophoresis in a 1.5% agarose gel. Appropriate bands were purified using the Clean-Up kit (A&A Biotechnology, Gdańsk, Poland), according to the manufacturer’s protocol, and sequenced by Macrogen Europe (Amsterdam, The Netherlands).

### 4.5. Chemical Analysis of Samples

Subsamples were mixed to contain one composite sample for the determination of pH in H_2_O and KCl by using pH/Jonometr CPI-502 (Elmetron, Zabrze, Poland) (pH determination by potentiometry, precision ±0.002 pH) and total Kjeldahl nitrogen [59] using Büchi Distillation Unit K-355 (Büchi, Flawil, Switzerland) (recovery rate ≥99.5%, reproducibility (RSD) ≤1%, detection limit ≥0.1 mg nitrogen). The total content of P and K in the samples was determined using the Egner–Riehm method [60]. For this purpose, for P using Thermo Helios Aquamate 9423 AQA 2000E (Thermo Electron Corporation, Altrincham, UK) and K BWBXP flame photometers (BWB Technologies, Newbury, Berkshire, UK) (specificity/K/ = <1% to each other when equal in concentration at <100 ppm), limit of detection (LOD K—0.02 ppm), and limit of quantification (LOQ K—0.05 ppm). In turn, the organic carbon (OC) was determined using a commercial Behr C50 IRF carbon analyzer (Labor-Technik GmbH, Dusseldorf, Germany) and the humus (organic matter) content in the samples was calculated using the conversion factor 1.724 [61].

### 4.6. Data Analysis

The obtained ITS sequences were analysed using BioEdit Sequence Alignment Editor (http://www.ncbi.nlm.nih.gov/, accessed on 2 June 2026). Using the BLAST algorithm, the sequences were compared with sequences deposited in the GenBank database of the National Center for Biotechnology Information (NCBI; http://www.ncbi.nlm.nih.gov/). The following criteria were used for taxonomic interpretation of ITS sequences: for sequence identity ≥97%, both genus and species names were accepted; for sequence identity between 95% and 97%, only the genus name was accepted; and for sequence identity <95%, the isolate was assigned to a higher taxonomic level or treated as unassigned [62]. The obtained sequences were deposited in the GenBank database.

To determine the species diversity of fungi at specific study sites, the Shannon Diversity Index (H) was used. It was calculated using the following equation: H = −∑ Pi(lnPi), where Pi stands for the proportion of each species in the sample [63].

To account for the high variance in fungal abundance across the sampling locations, spatial differences in culturable fungal counts (CFU·10^2^ g^−1^) from the soil and sediment samples of the Demänovská Ice Cave were normalized and evaluated using standardized values (*z*-scores). The z-scores were calculated to determine the distance of each individual sample value from the group mean in units of standard deviation, allowing for a standardized comparison of fungal proliferation across distinct thermal regimes and microhabitats [64].

The Pearson (*r*) correlation coefficient at α = 0.05 was used to determine the relation between the mean levels of N, P, K, pH and C/organic matter in the soil/sediments and the total concentration of fungi.

## 5. Conclusions

This study demonstrates a clear physiological boundary between psychrophilic cave-adapted fungal strains and mesophilic surface contaminants in the Demänovská Ice Cave. Under psychrophilic conditions (5 °C), subterranean sediments support proliferation of specialized, cold-tolerant fungi (such as *C. cladosporioides* and *Pseudogymnoascus* spp.) independently of localized nutrient concentrations. On the other hand, exposure to the mesophilic threshold (24 °C) triggers a severe biomass and biodiversity collapse inside the cave, confirming that true subterranean fungal specialists are inhibited by elevated temperatures. Furthermore, while fungal development in the outdoor environment is dictated by substrate richness, demonstrating statistically significant positive correlations with total nitrogen, available potassium, and organic matter, these relationships are completely dissociated within the cave system. This highlights the evolutionary capacity of specialized cave mycobiota to survive and colonize under extreme oligotrophic constraints. Also, these findings emphasize that subterranean microclimatic stability, rather than nutrient abundance, likely determines the ecological dynamics of ice cave mycobiota. Last but not least, species posing potential threats to human health were identified, including opportunistic and potentially azole-resistant pathogens (*A. fumigatus*, *A. tubingensis*), the dermatophytes (*P. pannorum*), allergenic bioaerosol components (*Cladosporium* and *Penicillium*), and toxigenic molds (*Penicillium*).

## Figures and Tables

**Table 1:** This Taxonomic classification, molecular identification metrics, and characterization of fungal isolates recovered from soil and sediment samples in the Demänovská Ice Cave across different incubation temperatures. All BLAST queries achieved 100% query coverage against reference sequences in the GenBank database. Fungal presence at a specific thermal threshold is indicated by a plus sign (+).

Identified Species	Isolate No.	Isolation Temperature		GenBank Accession No.	Sequence Length (bp)	Reference Sequence from GenBank	
		5 °C	24 °C			Accession No.	Identity (%)
*Akanthomyces muscarius*	UWR_1170	+	+	PZ770540	503	MT498092.1	100.00
*Aspergillus fumigatus*	UWR_1171		+	PZ770541	529	MT591987.1	100.00
*Aspergillus tubingensis*	UWR_1172		+	PZ770542	523	MK108401.1	99.43
*Cladosporium cladosporioides*	UWR_1173	+		PZ770543	355	OR251922.1	100.00
*Cosmospora viridescens*	UWR_1174	+	+	PZ770544	479	OR761556.1	100.00
*Juxtiphoma eupyrena*	UWR_1175	+		PZ770545	484	MK907745.1	100.00
*Linnemannia elongata*	UWR_1176	+		PZ770546	423	OQ726141.1	99.05
*Mortierella alpina*	UWR_1177	+	+	PZ770547	582	MT453274.1	100.00
*Oidiodendron truncatum*	UWR_1178	+		PZ770548	443	MW015991.1	100.00
*Penicillium camemberti*	UWR_1179		+	PZ770549	485	MT530220.1	100.00
*Penicillium chrysogenum*	UWR_1180	+	+	PZ770550	411	MT601877.1	100.00
*Penicillium* *commune*	UWR_1181		+	PZ770551	520	KC009786.1	100.00
*Penicillium echinulatum*	UWR_1182	+		PZ770552	480	KU325035.1	100.00
*Penicillium griseofulvum*	UWR_1183		+	PZ770553	477	MN545450.1	100.00
*Penicillium restrictum*	UWR_1184	+	+	PZ770554	460	MN900648.1	100.00
*Penicillium swiecickii*	UWR_1185	+		PZ770555	510	MN833349.1	100.00
*Pseudogymnoascus pannorum*	UWR_1186	+		PZ770556	468	MH859889.1	100.00
*Pseudogymnoascus roseus*	UWR_1187	+		PZ770557	387	MT477869.1	100.00
*Trichoderma harzianum*	UWR_1188	+	+	PZ770558	381	MT626719.1	100.00

**Table 2:** Physicochemical properties, mineral nutrient concentrations, and organic matter content of soil and sediment substrates across individual study sites of the Demänovská Ice Cave. Study site I represents the outdoor control location, whereas study sites II through VII represent the subterranean sampling zones inside the cave.

Study Sites	Total N	Total P	Total K	pH		% OC	% Organic Matter
	g·kg^−1^	mg·kg^−1^		in H_2_O	in KCl		
I	1.84	20.60	90	8.47	7.82	5.02	8.65
II	0.48	19.24	45	8.35	8.08	0.15	0.26
III	0.56	29.38	20	8.32	8.53	0.33	0.57
IV	0.44	28.77	45	8.49	7.99	0.49	0.84
V	0.20	34.81	35	8.37	7.97	0.86	1.48
VI	0.34	30.32	45	8.36	7.94	1.83	3.15
VII	1.02	41.53	35	8.62	7.99	4.03	6.95
Average (from II to VII)	0.51	30.68	37.50	8.42	8.08	1.28	2.21

## Data Availability

Experimental data will be made available upon request. The fungal ITS rDNA nucleotide sequences obtained in the study were submitted to GenBank under accession numbers PZ770540-PZ770558.

## Appendix A

**Table A1:** Descriptive statistics and standardized values (*z*-scores) for fungal CFU Count in soil or sediment samples of the Demänovská Ice Cave. Sampling location I represents the outdoor surface control site, whereas locations II through VII represent subterranean sampling zones inside the cave. Fungal abundance parameters are established at a baseline mean (M) of 68.6 CFU·10^2^ g^−1^ and a standard deviation (SD) of 75.5 CFU·10^2^ g^−1^.

Study Sites	Fungal Abundance (CFU·10^2^ g^−1^)	Deviation from Mean (DFM) (CFU·10^2^ g^−1^)	*z*-Score	Percentile	Interpretation
I	68.0	−0.6	−0.01	49.7%	Close to average
II	215.0	146.4	1.94	97.4%	Well above average
III	5.0	−63.6	−0.84	20.0%	Slightly below average
IV	10.0	−58.6	−0.78	21.9%	Slightly below average
V	118.0	49.4	0.66	74.4%	Slightly above average
VI	25.0	−43.6	−0.58	28.2%	Slightly below average
VII	39.0	−29.6	−0.39	34.7%	Slightly below average

**Table A2:** Descriptive statistics and standardized values (*z*-scores) for fungal CFU Count in soil or sediment samples of the Demänovská Ice Cave. Sampling location I represents the outdoor surface control site, whereas locations II through VII represent subterranean sampling zones inside the cave. Fungal abundance parameters are established at a baseline mean (M) of 19.6 CFU·10^2^ g^−1^ and a standard deviation (SD) of 37.17 CFU·10^2^ g^−1^.

Study Sites	Fungal Abundance (CFU·10^2^ g^−1^)	Deviation from Mean (DFM) (CFU·10^2^ g^−1^)	*z*-Score	Percentile	Interpretation
I	103.6	84.0	2.26	1.0	Substantially above average
II	2.9	−16.7	−0.45	0.3	Slightly below average
III	9.2	−10.4	−0.28	0.4	Slightly below average
IV	0.3	−19.3	−0.52	0.3	Slightly below average
V	6.4	−13.2	−0.36	0.4	Slightly below average
VI	5.0	−14.6	−0.39	0.3	Slightly below average
VII	10.0	−9.6	−0.26	0.4	Slightly below average

**Table A3:** Descriptive statistics and standardized values (*z*-scores) for fungal CFU Count in soil or sediment samples of the Demänovská Ice Cave. Sampling location I represents the outdoor surface control site, whereas locations II through VII represent subterranean sampling zones inside the cave. Fungal abundance parameters are established at a baseline mean (M) of 88.2 CFU·10^2^ g^−1^ and a standard deviation (SD) of 83.23 CFU·10^2^ g^−1^.

Study Sites	Fungal Abundance (CFU·10^2^ g^−1^)	Deviation from Mean (DFM) (CFU·10^2^ g^−1^)	*z*-Score	Percentile	Interpretation
I	171.6	83.4	1.00	84.2%	Well above average
II	217.9	129.7	1.56	94.0%	Well above average
III	14.2	−74.0	−0.89	18.7%	Slightly below average
IV	10.3	−77.9	−0.94	17.5%	Slightly below average
V	124.4	36.2	0.44	66.8%	Slightly below average
VI	30.0	−58.2	−0.70	24.2%	Slightly below average
VII	49.0	−39.2	−0.47	31.9%	Slightly below average

**Table A4:** Average concentration of culturable fungal species recovered from soil and sediment samples following incubation at 5 °C. Fungal counts are expressed as (CFU·10^2^ g^−1^). Sampling location I represents the outdoor surface control site, whereas locations II through VII represent subterranean sampling zones inside the Demänovská Ice Cave. Dashes (—) indicate that the specific fungal taxon was not detected.

Fungal Species	Study Sites						
	I ^1^	II	III	IV	V	VI	VII
*Akanthomyces muscarius*	8.0	—	1.0	—	—	—	—
*Cladosporium cladosporioides*	4.0	152.0	—	—	100.0	—	—
*Cosmospora viridescens*	2.0	—	—	—	—	—	—
*Juxtiphoma eupyrena*	9.0	25.0	—	—	4.0	2.0	—
*Linnemannia elongata*	1.0	1.0	—	—	—	3.0	1.0
*Mortierella alpina*	1.5	2.0	—	—	—	2.0	—
*Oidiodendron truncatum*	—	—	—	—	2.0	2.0	—
*Penicillium chrysogenum*	2.5	—	—	—	—	—	—
*Penicillium echinulatum*	—	—	—	—	—	1.0	—
*Penicillium restrictum*	14.0	—	—	—	10.0	—	—
*Penicillium swiecickii*	11.0	—	—	—	—	—	1.0
*Pseudogymnoascus pannorum*	3.0	20.0	2.5	10.0	2.0	8.0	24.0
*Pseudogymnoascus roseus*	2.0	15.0	1.5	—	—	2.0	3.0
*Trichoderma harzianum*	10.0	—	—	—	—	—	—

**Table A5:** Average concentration of culturable fungal species recovered from soil and sediment samples following incubation at 24 °C. Fungal counts are expressed as (CFU·10^2^ g^−1^). Sampling location I represents the outdoor surface control site, whereas locations II through VII represent subterranean sampling zones inside the Demänovská Ice Cave. Dashes (—) indicate that the specific fungal taxon was not detected.

Fungal Species	Study Sites						
	I ^1^	II	III	IV	V	VI	VII
*Akanthomyces muscarius*	—	—	—	—	1.4	—	5.0
*Aspergillus fumigatus*	3.0	—	—	—	—	—	—
*Aspergillus tubingensis*	—	—	—	—	—	—	2.0
*Cladosporium cladosporioides*	20.0	0.1	—	—	—	1.7	—
*Cosmospora viridescens*	—	2.6	9.0	0.3	4.0	1.0	1.0
*Mortierella alpina*	30.3	—	—	—	—	0.3	—
*Penicillium camemberti*	1.0	—	—	—	—	—	1.0
*Penicillium chrysogenum*	20.0	0.2	0.2	—	—	2.0	1.0
*Penicillium commune*	—	—	—	—	1.0	—	—
*Penicillium griseofulvum*	45.0	—	—	—	—	—	—
*Penicillium restrictum*	4.0	—	—	—	—	—	—
*Trichoderma harzianum*	0.3	—	—	—	—	—	—
